# Psychosexual Functioning Outcome Testing after Hypospadias Repair

**DOI:** 10.3390/healthcare8010032

**Published:** 2020-02-05

**Authors:** Marko Majstorovic, Marta Bizic, Dejan Nikolic, Borko Stojanovic, Marko Bencic, Ivana Joksic, Miroslav Djordjevic

**Affiliations:** 1 University Children’s Hospital, Tirsova 10, 11000 Belgrade, Serbiambencic88@gmail.cm (M.B.);; 2 Faculty of Medicine, University of Belgrade, 11000 Belgrade, Serbia; 3 Clinic for Gynecology and Obstetrics “Narodni front”, 11000 Belgrade, Serbia

**Keywords:** hypospadias, children, adults, long-term follow-up, psychosexual development, sexual functioning, Global Sexual Functioning questionnaire

## Abstract

Self-confidence plays an important role in both genders’ sexual functioning. Lack of genital self-esteem may have negative effects on psychosexual development, especially in males, where deviations from a standardized normal penile appearance can lead to inhibitions in entering into sexual relationships. The aim of our study was to evaluate the informativeness of studied domains of the Global Sexual Functioning (GSF) questionnaire and sexual functioning of patients surgically treated in childhood for different types of hypospadias. We evaluated 63 males with hypospadias and 60 healthy age- and gender-matched controls. The GSF questionnaire was used to estimate psychosexual function as a long-term follow-up after the surgical correction of hypospadias in the patient and control groups. Sexual activity (*p* = 0.017), arousal (*p* = 0.033) and orgasmic abilities (*p* = 0.002) values were significantly increased in patients. Strong correlation was noticed between sexual activity and sexual desire (R = 0.872); arousal and sexual desire (R = 0.753), as well as orgasmic and erectile abilities (R = 0.769). Different domains of psychosexual functioning in the patient group correlated with each other to various degrees, resulting in a heterogeneous expression of psychosexual dysfunctions, implicating the necessity of a personalized treatment approach.

## 1. Introduction

Urogenital congenital anomalies are among the most common congenital anomalies in the pediatric population, with hypospadias being one of the most frequent with the incidence of 1 in every 200–300 male livebirths [1]. Hypospadias is characterized by an ectopic ventrally-positioned urethral meatus, ventral chordee, and excessive dorsal foreskin [2].

To date, there have been over 300 different surgical techniques and their modifications for hypospadias repair, and still hypospadias repair poses a great challenge even for experienced pediatric surgeons and urologists. The goal of each of these techniques is to repair all present anomalies, involving urethral reconstruction, correction of any penile curvature, glans reconstruction and penile skin reconstruction, creating a functional and esthetically appealing penis [3]. It is advised that surgeries should be performed in an early age (until the age of two years of life), as it is presumed that genital awareness has not been established at that time and that the anxiety of separation from the mother will be diminished [1,4,5,6].

Puberty brings important physical and psychological changes in every child’s life. As the genitals start to grow visibly, sexual function and esthetics become important issues for every boy [7,8]. Sometimes, initially successful hypospadias repair turns out to have one of the postoperative complications that can have an impact on psychosexual development of the patient [9]. The majority of studies dealing with hypospadias repair report high success rates, but there are those that report patients’ dissatisfaction regarding the appearance and size of their penises compared to men without hypospadias [10,11,12,13]. Few studies report the long-term results of hypospadias treated in childhood and their influence on sexual and psychosexual functioning [9,14,15]. These reports are somewhat contradictory, as some authors found hypospadias patients to be less sexually active than healthy controls, while others reported similar psychosexual development as in controls [9,14,16].

Self-confidence plays an important role in male and female sexual functioning. Individuals who lack self-esteem regarding their genitals may experience negative effects on psychosexual development, with cross-gender behavior and reversed gender roles. This is especially present in male population, where deviations from a standardized normal penile appearance can lead to inhibitions in entering into sexual relationships [17].

The Global Sexual Functioning questionnaire is a multidimensional questionnaire evaluating different levels of psychosexual functioning in males and females [18]. In order to evaluate the sexual functioning more broadly, we made the hypothesis that the previously proposed GSF questions in the defined domains have lower intraclass correlation, which will enable the examiner to obtain more information for each of the aforementioned domains. 

Therefore, the aim of our study was twofold—first to evaluate the informativeness of the studied domains of GSF, and second to investigate the sexual functioning of patients surgically treated in childhood for different types of hypospadias and to compare them with healthy controls. 

## 2. Material and Methods

### 2.1. Study Group

A total of 286 male patients were surgically treated for isolated hypospadias by board-certified pediatric urologists in our center between 1992 and 2002. Participants and their parents/legal guardians were contacted by phone call and/or email (where applicable) in order to participate in long-term follow-up study. Participation in the study was accepted by 78 (27.27%) patients, from which 63 (80.77%) fulfilled the inclusion criteria (normal range of sex-hormones, no report on psychiatric disorders verified by board-certified psychiatrists, control check-up and returned completely filled questionnaire). Hypospadias patients were grouped into distal (41 patient), midshaft (10 patients), penoscrotal (9 patients), and scrotal (3 patients) [19]. Patients with associated congenital genital anomalies (cryptorchidism, inguinal hernia, disorder of sexual development) and other congenital malformations were excluded from the study. The survey was conducted between January 2017 and June 2018, with individuals at least 16 years old at the time of survey. Mean age of patients included in our study was 21.79 ± 2.40 years (ranging from 16 to 26 years). The control group at the time of survey was age 22.02 ± 2.81 years, range from 16.5 to 28 years, and gender (male) matched. 

The control group of healthy male individuals was created by randomly selecting 60 charts of patients at our medical facility. Board-certified urologists and pediatric surgeons examined the patients before the interview and excluded all genital anomalies or genital surgeries. 

None of patients nor controls from our study, at the time of survey, were married, however, they were all sexually active.

Prior to the inclusion in the study, patients or parents/legal guardians were informed of the study protocol and informed consent was obtained. The study was approved by Institutional Review Board and followed the principles of good clinical practice.

### 2.2. Testing Instrument

Global Sexual Functioning score (Supplementary Material Table S1) concisely and accurately reflects the overall level of sexual functioning. It is calculated using 12 of the 46 items of the Sexual History Form (SHF). The 12 items were selected as representative of various domains of sexual functioning: frequency of sexual activities (item 1 and item 2), sexual desire (item 6 and item 7), arousal (item 10 and item 16), erectile abilities (item 18, item 19 and item 22), and orgasmic abilities (item 23, item 24, and item 25) [18]. The single summary score is derived by 1) converting the scores on each of the 12 items to a fraction of the maximum possible value; 2) summing up the 12 fractions; and 3) calculating the mean by dividing the total by the number of items that the respondent is deemed to have answered. The Global Functioning Score varies between 0 and 1, with lower scores signifying better functioning [18]. We followed the forward-backward method for translation of the GSF questionnaire from English into Serbian as well as principles of framework for translation and cultural adaptation considering patient-reported outcome parameters [20,21,22]. 

### 2.3. Statistical Analysis

For the estimation of the statistical difference between controls and patients for every item in every domain, as well between domains, we used the Mann Whitney U test. To evaluate the association between questions and how they resemble each other in certain domains, we performed the Intraclass correlation (ICC). ICC values were graded as: <0.40 poor, between 0.40–0.59 fair; between 0.60–0.74 good, and between 0.75–1.00 excellent [23]. To evaluate the variability between the questions tested in a single domain we used η^2^ (%). Pearson’s correlation was used to assess the degree of correlation between different domains of the GSF questionnaire of the patient group. Statistical significance was set at *p* < 0.05. Data analyses were performed in SPSS 20 (IBM Corporation, Armonk, NY, USA) statistical software.

## 3. Results

### 3.1. Tested Parameters and Characteristics of Study Group

Table 1 presents the general characteristics of the tested patients that were surgically treated for hypospadias.

Complications that occurred in the group of patients were divided into early—5 (62.50%) patients (one patient with midshaft hypospadias developed a urinary tract infection that was treated with antibiotics; one patient with scrotal hypospadias developed urethral fistula that required minor surgical repair; three patients developed meatal stenosis: one patient with distal hypospadias, one patient with midhsaft hypospadias, and one patient with penoscrotal hypospadias that was repaired by meatoplasty). Mean age of late complication onset was 13.67 ± 1.76 years. Late complications occurred in 3 (37.50%) patients: meatal stenosis developed in one patient with distal hypospadias and was conservatively solved; urethral stenosis developed in one patient with midshaft hypospadias and required augmentation urethroplasty with buccal mucosa graft; and urethral diverticulum developed in one patient with penoscrotal hypospadias and required excision and urethroplasty. At the time of the inclusion in the study, none of our patients were with residual complications.

### 3.2. Informativeness of Studied Domains of the Global Sexual Functioning Questionnaire

There is a poor correlation between the tested questions both for controls and patients for sexual desire, arousal, and orgasmic abilities, while for frequency of sexual activities correlation was poor in controls and fair for patients (Table 2). Regarding erectile abilities, there was a fair correlation for controls and an excellent correlation for patients, with excellent correlation between GSF 18/19. (Table 2).

### 3.3. Sexual Functioning of Surgically Treated Patients with Hypospadias 

In the patient group, scores of all questions were increased compared to controls, but there was a significant increase in the value of GSF 1 (*p* = 0.027), GSF 16 (*p* = 0.035), GSF 19 (*p* = 0.038), and GSF 25 (0.012), and a highly significant increase in the values of GSF 23 (*p* = 0.012) (Table 3).

In the patient group, scores of all domains were increased compared to controls, but there was a significant increase in the value for frequency of sexual activities (*p* = 0.017) and arousal (0.033), and a highly significant increase in the values for orgasmic abilities (*p* = 0.002) (Table 4).

For the frequency of sexual activities, there was a strong positive correlation with sexual desire (R = 0.872) and moderate positive correlation with arousal (R = 0.617), erectile abilities (R = 0.675), and orgasmic abilities (R = 0.643). For sexual desire, there was a strong positive correlation with arousal (R = 0.753), erectile abilities (0.528), and orgasmic abilities (0.654). For arousal, there was a weak positive correlation with erectile abilities (R = 0.235) and moderate positive correlation with orgasmic abilities (R = 0.515). For erectile abilities, there was a strong positive correlation with orgasmic abilities (R = 0.769) (Table 5).

Surgically treated patients with distal hypospadias were with the lowest values for all domains, while those that were surgically treated with scrotal hypospadias had the highest values for all domains (Table 6).

## 4. Discussion

Hypospadias is the most common congenital anomaly of the urogenital tract in males, with an incidence of 1 in every 200–300 male livebirths. It is usually corrected within the first two years of life [1,5]. Psychosexual development ends after puberty, thus psychological and sexual function of patients surgically treated for hypospadias can be evaluated only after puberty [8]. According to some studies, this development can be impaired if there are functional or esthetic problems with the penis due to hypospadias correction. Studies reporting on the long-term follow-up of hypospadias are relatively rare, with a small patient sample, which can be explained by difficulties in approaching patients several years or decades after the primary hypospadias repair [9]. So far, several questionnaires were developed and used in the evaluation of hypospadias repair outcome. However, there is still a lack of standardized questionnaires for psychosexual function evaluation available in literature [19,24]. Therefore, we evaluated the long-term outcome with a GSF questionnaire to add more information about the specific components of psychosexual improvement after hypospadias repair in males to the available literature.

Different domains of the GSF questionnaire in our study on patients with hypospadias have various degrees of informativeness owing to the fact that selected items in studied domains are selective and bring about a more complete evaluation of psychosexual function in these patients. However, we have noticed that for the erectile abilities domain, the narrowest range of informativeness is obtained with the items dealing with getting and keeping an erection before and during intercourse. Considering sexual functioning, our findings are in line with the aforementioned statements, particularly in terms of frequency of sexual activities, arousal, and erectile abilities. Despite the fact that we found a nonsignificant difference between controls and patients in the erectile abilities domain, it should be stressed that GSF item 19 showed a significant increase. To a certain degree this is in line with previous findings of Jiao et al., who stated that the majority of hypospadias patients in their study reported good erection ability [25]. However, assuming multidimensional aspects of the male erection (to achieve, maintain, and to climax), further studies are advised since we have demonstrated that for certain aspects (to maintain erection) other factors could play a role in a proper erection response and climax. 

One of the first studies that comprised 213 patients treated for hypospadias showed the need for the long-term follow-up of this group of patients, as 72% of the examinees reported that a normal appearance of the penis was important for normal sexual function [17]. The success rate of hypospadias repair depends on different factors, such as severity of hypospadias, age at the correction, one or two-stage repair, prior failed repairs, as well as a lack of material for reconstruction of all penile deformities. Mureau et al. [11] found that patients with proximal hypospadias were less satisfied than those with distal forms. In these patients, ventral curvature was always present, and its correction with the plication technique usually affected the already compromised penile size. They also emphasized that patients with hypospadias feared that absence of the prepuce would reveal the anomaly, because circumcision was not common in their culture [11]. The same findings were discussed in a study of Jones et al. as the idea of what would be considered cosmetically normal is vastly different among patients, surgeons, and cultures [26]. The satisfaction with genital appearance is correlated with psychosexual development, as described by Jones et al. and Mureau et al. [26,27]. Furthermore, different aspects of genital appearance might affect each other, making the final outcome more or less challenging [25]. In our study, we have shown that different domains correlate with each other with different degrees. This clearly shows that different impairments of different domains on psychosexual level might influence other studied domains as well. Therefore, despite the fact that the approach to each patient should be individual, it is also important to analyze and monitor each component of psychosexual function to achieve the best overall outcome. 

Literature is also missing comprehensive follow-up studies of patients initially successfully treated for hypospadias in childhood, as the majority of these patients are lost to follow-up. One possible hypothesis for such dispersion could be that as patients reach maturity, the psychosexual function gets less challenging. Furthermore, Jiao et al. addressed that these patients, after being sexually mature, usually do not stay at the same place or refuse the participation in long-term evaluation studies regarding the treatment of hypospadias [25]. Having said that, our response rate of eligible participants (22.03%) was similar with the response rate of previous studies dealing with long-term results of hypospadias treatment [24,25]. The patients get socialized and incorporated into the community and thus gain better independence and social awareness. Our results are consistent with such claims, as there were inverse predictive trends between age and functional scores for all domains, meaning that as individuals age, they show lower scores. However, for the majority of centers, it is still difficult to get a real insight into long-term postoperative results and complications [13]. These patients usually know little about the initial position of the meatus and associated anomalies, as well as the surgical approach. They represent a complex clinical population that requires interaction between pediatric and adult urologists [28]. Transitional urology tends to connect the pediatric and adult urologists to provide continuous monitoring of patients treated for hypospadias and other congenital urogenital anomalies that require long-term follow-up [29,30]. It seems that all patients with various types of hypospadias in our study population had the highest scores for the frequency of sexual activity domain, which proves the presence of impaired intention in seeking sexual contacts when compared to healthy controls. Our findings are consistent with previous reports where proximal hypospadias showed less favorable scores in tested domains of the GSF questionnaire. Furthermore, the most challenging type of proximal hypospadias in our study was scrotal hypospadias, with the highest increase in values for all domains. Patients with scrotal hypospadias were more constrained for willingness and desire for sexual contact compared to other patient groups and healthy controls, which is in agreement with previous studies [11,31].

There are several limitations to this study. The first limitation is the single population, where specific pleiotropic variations in psychosocial and psychosexual domains might exist in different populations, and thus a multipopulational approach should be envisaged for future studies. Further, the present number of studied patients could be considered as a limiting factor. Therefore, a larger sample would be suggested for more representative findings.

## 5. Conclusions

Despite the fact that the study sample could be taken to a certain degree as a limitation factor, we have demonstrated that different domains of the GSF questionnaire have satisfactory informativeness regarding the complete estimation of sexual functioning in studied subjects. Furthermore, we have shown that for patients who underwent surgical correction of hypospadias, psychosexual functioning differed compared to age- and gender-matched healthy individuals. Different domains of such functioning might be affected to different degrees. Moreover, we have shown that different domains correlate with each other to various degrees, resulting in heterogeneous expression of psychosexual functioning. However, it should be stated as well that different types of hypospadias might result in various degree of sexual dysfunction. This shows the necessity of a personalized approach in treatment along with follow-up of these patients that will include psychological counseling of adolescents and young adults. Such an approach will ultimately achieve better socialization, increased self-awareness, and optimal overall quality of the patients’ lives.

## Figures and Tables

**Table 1:** General characteristics of the tested patients.

Parameters			Values
Hypospadias Type		Distal, n (%)	41 (65.08)
		Midshaft, n (%)	10 (15.87)
		Penoscrotal, n (%)	9 (14.29)
		Scrotal, n (%)	3 (4.76)
Curvature Present		Distal, n (%)	10 (24.39)
		Midshaft, n (%)	8 (80.00)
		Penoscrotal, n (%)	9 (100.00)
		Scrotal, n (%)	3 (100.00)
Age at the time of surgery (months, MV ± SD)			20.76 ± 6.41
Age at the time of testing (years, MV ± SD)			21.79 ± 2.40
Surgical Techniques	Distal	Tubularized incised-plate urethroplasty, n (%)	6 (14.63)
		Tubularized incised-plate urethroplasty with dartos flap, n (%)	35 (85.37)
	Midshaft	Tubularized incised-plate urethroplasty with dartos flap, n (%)	10 (100.00)
	Penoscrotal	Skin flap urethroplasty, n (%)	6 (66.67)
		Buccal mucosa graft + skin flap urethroplasty, n (%)	3 (33.33)
	Scrotal	Buccal mucosa graft + skin flap urethroplasty, n (%)	3 (100.00)
Complications		For distal hypospadias repair, n (%)	2 (4.88)
		For midshaft hypospadias repair, n (%)	3 (30.00)
		For penoscrotal hypospadias repair, n (%)	2 (22.22)
		For scrotal hypospadias repair, n (%)	1 (33.33)

MV, mean value; SD, standard deviation.

**Table 2:** Intraclass correlation values for defined domains in controls and patients.

Domains	Controls		Patients			
	ICC (95% CI)	η^2^ (%)	ICC (95% CI)			η^2^ (%)
Frequency of sexual activities	0.287 (0.038–0.501)	63.93	0.457 (0.239–0.631)			72.52
Sexual desire	0.101 (−0.154–0.344)	54.64	0.334 (0.097–0.535)			66.34
Arousal	0.072 (−0.181–0.318)	53.23	0.158 (−0.089–0.389)			57.55
Erectile abilities	0.516 (0.261–0.732)	75.60	0.897	GSF 18/19	0.897 (0.834–0.936)	94.72
				GSF 18/22	0.411 (0.185–0.597)	
				GSF 19/22	0.398 (0.169–0.586)	
Orgasmic abilities	0.084 (−0.409–0.422)	45.39	0.273 (0.150–0.468)			63.24

CI, confidence interval; ICC, intraclass correlation.

**Table 3:** Values and differences between domains of controls and patients.

Domains	Questions	Controls N = 60		Patients N = 63		*p* *
		MV ± SD	95% CI	MV± SD	95% CI	
Frequency of sexual activities	GSF 1	0.40 ± 0.14	0.36–0.44	0.52 ± 0.25	0.46–0.58	0.027 ^†^
	GSF 2	0.33 ± 0.14	0.29–0.37	0.38 ± 0.19	0.34–0.43	0.147
Sexual desire	GSF 6	0.23 ± 0.16	0.19–0.27	0.28 ± 0.23	0.23–0.34	0.384
	GSF 7	0.46 ± 0.25	0.39–0.53	0.48 ± 0.26	0.42–0.55	0.631
Arousal	GSF 10	0.32 ± 0.25	0.26–0.38	0.35 ± 0.23	0.29–0.40	0.308
	GSF 16	0.28 ± 0.12	0.25–0.31	0.38 ± 0.23	0.32–0.44	0.035 ^†^
Erectile abilities	GSF 18	0.25 ± 0.10	0.22–0.27	0.32 ± 0.19	0.27–0.37	0.159
	GSF 19	0.26 ± 0.13	0.22–0.29	0.34 ± 0.20	0.29–0.39	0.038 ^†^
	GSF 22	0.41 ± 0.28	0.34–0.49	0.44 ± 0.23	0.38–0.49	0.171
Orgasmic abilities	GSF 23	0.23 ± 0.12	0.20–0.26	0.35 ± 0.20	0.30–0.40	0.001 ^†^
	GSF 24	0.40 ± 0.29	0.33–0.48	0.45 ± 0.19	0.40–0.50	0.083
	GSF 25	0.22 ± 0.06	0.20–0.23	0.28 ± 0.12	0.25–0.31	0.012 ^†^

* Mann Whitney U test; ^†^ Statistically significant; CI, confidence interval; GSF, Global Sexual Functioning.

**Table 4:** Values and differences between domains of controls and patients.

Domains	Controls		Patients		*p* *
	MV ± SD	95% CI	MV ± SD	95% CI	
Frequency of sexual activities	0.36 ± 0.12	0.33–0.40	0.46 ± 0.20	0.41–0.51	0.017 ^†^
Sexual desire	0.36 ± 0.20	0.31–0.41	0.39 ± 0.21	0.33–0.44	0.549
Arousal	0.30 ± 0.14	0.27–0.34	0.37 ± 0.17	0.32–0.41	0.033
Erectile abilities	0.31 ± 0.13	0.27–0.34	0.36 ± 0.18	0.32–0.41	0.119
Orgasmic abilities	0.29 ± 0.14	0.26–0.33	0.36 ± 0.14	0.33–0.40	0.002 ^†^

* Mann Whitney U test; ^†^ Statistically significant; CI, confidence interval.

**Table 5:** Correlations between different domains of GSF questionnaire in the patient group.

Domains *		Patients				
		Frequency of Sexual Activities	Sexual Desire	Arousal	Erectile Abilities	Orgasmic Abilities
**Patients**	Frequency of sexual activities	-	0.872	0.617	0.675	0.643
	Sexual desire	-	-	0.753	0.528	0.654
	Arousal	-	-	-	0.235	0.515
	Erectile abilities	-	-	-	-	0.769
	Orgasmic abilities	-	-	-	-	-

* Pearson’s correlation.

**Table 6:** GSF scores in patients with different types of hypospadias.

Domains	Distal Hypospadias MV ± SD (95% CI)	Midshaft Hypospadias MV ± SD (95% CI)	Penoscrotal Hypospadias MV ± SD (95% CI)	Scrotal Hypospadias MV ± SD (95% CI)
Frequency of sexual activities	0.42 ± 0.15 (0.37–0.46)	0.46 ± 0.19 (0.33–0.60)	0.47 ± 0.25 (0.26–0.64)	0.72 ± 0.25 (0.09–1.35)
Sexual desire	0.34 ± 0.17 (0.29–0.40)	0.36 ± 0.19 (0.22–0.50)	0.37 ± 0.25 (0.17–0.56)	0.72 ± 0.20 (0.24–1.21)
Arousal	0.32 ± 0.17 (0.29–0.39)	0.33 ± 0.16 (0.20–0.45)	0.40 ± 0.21 (0.25–0.55)	0.56 ± 0.07 (0.40–0.72)
Erectile abilities	0.32 ± 0.19 (0.22–0.41)	0.34 ± 0.14 (0.23–0.44)	0.40 ± 0.22 (0.22–0.57)	0.61 ± 0.22 (0.06–1.16)
Orgasmic abilities	0.34 ± 0.12 (0.31–0.38)	0.35 ± 0.16 (0.23–0.47)	0.35 ± 0.15 (0.23–0.47)	0.49 ± 0.10 (0.24–0.74)

GSF, Global Sexual Functioning; MV, mean vale; SD, standard deviation.

## Supplementary Materials

The following are available online at https://www.mdpi.com/2227-9032/8/1/32/s1, Table S1: Global Sexual Functioning Score (short form of the original 46-item Sexual History Form (SHF) Questionnaire).
