# Supplementary material for: Psychosexual Functioning Outcome Testing after Hypospadias Repair
Source: Healthcare (Basel). 2020 Feb 5;8(1):32. doi: 10.3390/healthcare8010032 (PMC7151077; doi:10.3390/healthcare8010032)
Supplement: Supplementary file 1 [file healthcare-08-00032-s001.pdf]

Supplementary material 1.
Table S1.

Global Sexual Functioning Score (short form of the original 46-item Sexual History Form (SHF) Questionnaire)

Please circle the most appropriate response to each question:

1. How frequently do you and your mate have sexual intercourse or activity?
   (Question 1 in SHF)
   1) More than once a day
   2) Once a day
   3) 3 to 4 times a week
   4) Twice a week
   5) Once a week
   6) Once every two weeks
   7) Once a month
   8) Less than once a momnth
   9) Not at all

2. How frequently you like to have sexual intercourse or activity?
   (Question 2 in SHF)
   1) More than once a day
   2) Once a day
   3) 3 to 4 times a week
   4) Twice a week
   5) Once a week
   6) Once every two weeks
   7) Once a month
   8) Less than once a momnth
   9) Not at all

3. How often do you experience sexual desire (this may iclude wanting to have sex, planning to have sex, feeling frustrated due to lack of sex, etc)?
   (Question 6 in SHF)
   1) More than once a day
   2) Once a day
   3) 3 to 4 times a week
   4) Twice a week
   5) Once a week
   6) Once every two weeks
   7) Once a month
   8) Less than once a momnth
   9) Not at all

4. How often do you masturbate (bring yourself to orgasm in private)?
   (Question 7 in SHF)
   1) More than once a day
   2) Once a day
   3) 3 to 4 times a week
   4) Twice a week
   5) Once a week
   6) Once every two weeks
   7) Once a month
   8) Less than once a momnth
   9) Not at all

5. Does the male ever reach orgasm while he is trying to enter the vagina with his penis?
   (Question 10 in SHF)
   1) Never
   2) Rarely (less than 10% of the time)
   3) Seldom (less than 25% of the time)
   4) Sometimes (50% of the time)
   5) Usually (75% of the time)
   6) Nearly always (over 90% of the time)

6. When you have sex with your mate do you feel sexually aroused (e.g. feeling „turned on“, pleasure, excitement)?
   (Question 16 in SHF)
   1) Nearly always (over 90% of the time)
   2) Usually (about 75% of the time)
   3) Sometimes (about 50% of the time)
   4) Seldom (about 25% of the time)
   5) Never

7. Does the make have any trouble getting an erection before intercourse begins?
   (Question 18 in SHF)
   1) Never
   2) Rarely (less than 10% of the time)
   3) Seldom (less than 25% of the time)
   4) Sometimes (50% of the time)
   5) Usually (75% of the time)
   6) Nearly always (over 90% of the time)

8. Does the male have any trouble keeping an erection once intercourse has begun?
   (Question 19 in SHF)
   1) Never
   2) Rarely (less than 10% of the time)
   3) Seldom (less than 25% of the time)
   4) Sometimes (50% of the time)
   5) Usually (75% of the time)
   6) Nearly always (over 90% of the time)

9. Does the male ejaculate (climax) without having a full, hard erection?
   (Question 22 in SHF)
   1) Never
   2) Rarely (less than 10% of the time)
   3) Seldom (less than 25% of the time)
   4) Sometimes (50% of the time)
   5) Usually (75% of the time)
   6) Nearly always (over 90% of the time)

10. If you try, is it possible to reach orgasm 9sensation of climax) through masturbation?
    (Question 23 in SHF)
    1)Nearly always (over 90% of the time)
    2) Usually (about 75% of the time)
    3) Sometimes (about 50% of the time)
    4) Seldom (about 25% of the time)
    5) Never
    6) Have never tried to

11. If you try, is it possible for you to reach orgasm (sensation of climax) through having yeour ghenitals caressed by your mate?
    (Question 24 in SHF)
    1)Nearly always (over 90% of the time)
    2) Usually (about 75% of the time)
    3) Sometimes (about 50% of the time)
    4) Seldom (about 25% of the time)
    5) Never
    6) Have never tried to

12. If you try, is it possible for you to reach orgasm (sensation of climax) through sexual intercourse?
    (Question 25 in SHF)
    1)Nearly always (over 90% of the time)
    2) Usually (about 75% of the time)
    3) Sometimes (about 50% of the time)
    4) Seldom (about 25% of the time)
    5) Never
    6) Have never tried to
